# Linking population dynamics models with empirically derived models through phytoplankton primary production

**DOI:** 10.1002/ece3.8339

**Published:** 2021-11-19

**Authors:** Motomi Genkai‐Kato

**Affiliations:** ^1^ Graduate School of Kuroshio Science Kochi University Kochi Japan

**Keywords:** cyanobacteria, phytoplankton, population dynamics, primary production

## Abstract

There are alternative methods for estimation of phytoplankton primary production (PP) that are fundamentally different in the calculation approach. The process‐oriented PP model is a mechanistic, empirically derived method based on the photosynthesis–light relationships. The population dynamics‐based PP calculation, which is a synthetic method, provides a production estimate based on population dynamics of phytoplankton. These alternative methods were here compared with regard to production estimates and linked to enhance the performance of the existing models of population dynamics applied to a wide variety of lakes worldwide in terms of morphometry, nutrient status, and light environments. Estimates of PP were shown to be sensitive to changes in phytoplankton sinking and zooplankton grazing rates in both methods. Production estimates in the process‐oriented PP model were also sensitive to light‐associated parameters such as day length. Although the production estimated from the population dynamics‐based PP calculation tended to be lower than that from the process‐oriented PP model irrespective of lake morphometry, production estimates calculated from both methods with standard parameterization were comparable when production was estimated on an annual timescale. However, it was also shown that the alternative methods could produce different production estimates when estimated on shorter timescales such as cyanobacterial blooms in summer. Cyanobacteria with low mortality due to grazing and sinking losses have been considered as trophic bottlenecks, but there is increasing evidence that their mortality is, to a considerable extent, due to parasitic pathogens. In the case of cyanobacterial blooms, an addition of parasite‐related loss term (19%–33% of standing stock) resulted in a resolution of the difference in production estimates between the methods. These analyses theoretically support the critical role of parasitism and resolve the bottleneck problem in aquatic ecosystem metabolism.

## Graphical abstract

Although the production estimated from the population dynamics‐based PP calculation (Method 2) tended to be lower than that from the process‐oriented PP model (Method 1) irrespective of lake morphometry, production estimates calculated from both methods with standard parameterization were comparable when production was estimated on an annual timescale. However, it was also shown that the alternative methods could produce different production estimates when estimated on shorter timescales such as cyanobacterial blooms in summer.

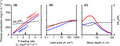

## 1 INTRODUCTION

In ecology, the modeling approach is diverse, ranging from simple, abstract models free of system‐specific details for understanding general principles to complex, simulation models for predicting the dynamics of specific systems. For the progress of ecological research, it is important to combine insights from both simple and complex models (Evans et al., 2013). Complex models are preferred for predictions about real ecological systems, but simple models have great utility in acting as submodels of more complex models. Simple models of population dynamics have contributed considerably to food web and ecosystem ecology. They are useful tools for analyzing predator–prey interactions, food webs, and ecosystem processes, and have provided important implications for ecosystem management (e.g., Beig & McCann, 2020; Beisner et al., 2003; Genkai‐Kato, 2007).

Simple models, as well as complex models, of food web and ecosystem models with multiple trophic levels usually consist of basal autotrophic organisms and their consumers. Primary production (PP) at the basal trophic level forms the base of food webs and is an important ecosystem process, because it is tied to the population dynamics of primary producers and consumers. Because PP is, by definition, the rate at which biomass is produced per unit area by autotrophic organisms, the growth term of autotrophs in population dynamics is closely linked to PP (Genkai‐Kato et al., 2012; Kelly et al., 2018). In other words, PP can be calculated from population dynamics at the basal trophic level (population dynamics‐based PP calculation).

In aquatic ecosystems, PP of phytoplankton is often estimated from a process‐oriented mechanistic model using the photosynthesis–light relationship (McBride, 1992; Phillips, 2020; Vadeboncoeur et al., 2008). In the process‐oriented PP model, the light condition is nonlinearly dependent on both time and depth such that the light varies sinusoidally (i.e., sine curves) from sunrise to sunset and decreases exponentially down from surface to bottom. Because of these spatiotemporal characteristics of the light condition, time and depth integrals or summations are needed to calculate PP.

The process‐oriented PP model is an empirically derived method where PP is calculated by integrating photosynthetic rates depending on spatiotemporal changes in light environments, while the population dynamics‐based PP calculation is rather a synthetic method where PP is implicitly included in the growth term of population dynamics of phytoplankton. These alternative methods that adopt fundamentally different approaches need to be compared and linked to enhance model performance of population dynamics, yet such a comparison has not been made in relation to limnological characteristics such as lake morphometry, nutrients and light environments. Lakes in nature have a large variation in size and nutrient status, and are distributed over the earth's surface. PP and photosynthesis of phytoplankton are dependent on lake morphometry (Carpenter, 1983), nutrient status (Schindler et al., 1973), and light environments (Kirk, 1994).

Here, I examine production estimates calculated from the alternative methods and identify parameters to which production estimates are sensitive, and discuss limnological characteristics that would lead to different and similar estimates. This paper suggests practical calibration, not a substantial alteration, of the existing simple models as submodels to enhance the performance of more complex models applied to a wide variety of lakes worldwide in terms of morphometry (e.g., from shallow to deep lakes), nutrient status (e.g., dominant phytoplankton), and light environments (e.g., season). Specifically, in the case of a lake dominated by cyanobacteria, which are less vulnerable to grazing and sinking losses, an additional source of mortality is integral to matching the methods, suggesting that parasitism plays an important role in the loss processes of cyanobacteria.

## 2 METHODS

### 2.1 Population dynamics at basal trophic level

Population dynamics of phytoplankton is composed of two dynamic variables in the epilimnion: phytoplankton density (*X*, mg‐chl·m^−1^) and phosphorus concentration (*N*, mg‐P·m^−1^):

(1) $$\frac{dX}{dt}=\frac{\mu N}{N+k}X‐\left(g+\frac{v}{z_{e}}+h\right)X,$$

(2) $$\frac{dN}{dt}=l+q_{P}egX‐\frac{q_{P}\mu X}{N+k}N‐hN.$$

I assume that the water is well mixed within the epilimnion so that the entire phytoplankton community is affected by the collective consumption of phosphorus throughout the epilimnion (Diehl, 2002). The meanings and units of the parameters are listed in Table 1. As is often the case with food‐web models of multiple trophic levels where growth rates are implicitly limited by a factor for analytical simplicity (e.g., Beisner et al., 2003), the growth of phytoplankton is here assumed to be primarily nutrient‐limited and described by the Monod function (Grover, 1989). The effect of light environments will be taken into consideration when PP is calculated by the process‐oriented PP model. Phytoplankton losses occur through grazing by zooplankton, sinking, and flushing from the lake. The rate of sinking loss is the sinking velocity of phytoplankton *v*, divided by the epilimnion thickness *z*_e_. The epilimnion thickness is calculated based on lake area *A* (Hanna, 1990):

(3) $$z_{e}=6.95A^{0.185}.$$

**TABLE 1:** Parameters and their units

Symbol	Meaning	Units	Value			Source
			Default	Minimum	Maximum	
*A*	Lake area	km^2^	10	0.1	1000	
*D*	Depth ratio	Dimensionless	0.5	0.33	0.67	Carpenter (1983)
*e*	Phosphorus release rate from dead phytoplankton	Dimensionless	0.5	0.4	0.8	Carpenter (1992), Grover (1995)
*g*	Zooplankton grazing rate	d^−1^	0.133	0.029	0.338	Gulati et al. (1982)
*h*	Flushing rate	d^−1^	0.001	0.01	0.0001	Cole and Pace (1998)
*I*_k_	Light intensity at onset of saturation	μmol·m^−2^·s^−1^	120	90	150	Reynolds (2006)
*I*_0_	Light intensity just below the water surface at noon	μmol·m^−2^·s^−1^	600	30	1200	Appendix S1
*k*	Half‐saturation constant for phytoplankton growth rate	mg‐P·m^−1^	4.5	1.1	10.9	Sommer (1989)
*L*	Areal phosphorus loading rate	mg‐P·m^−2^·d^−1^	2.3	0.26	4.6	Lathrop et al. (1998)
*l*	Volumetric phosphorus loading rate	mg‐P·m^−3^·d^−1^	= $L/\bar{z}$			
*P*_max_	Maximum photosynthetic rate of phytoplankton	mg‐C·mg‐chl^−1^·h^−1^	3.5	2.4	4.7	Megard (1972)
*q*_C_	Phytoplankton carbon content	mg‐C·mg‐chl^−1^	47	27	67	Riemann et al. (1989)
*q*_P_	Phytoplankton phosphorus content	mg‐P·mg‐chl^−1^	= 2.58 *q*_C_/θ			
*s*	Phytoplankton shading attenuation coefficient	m^2^·mg‐chl^−1^	0.021	0.007	0.066	Genkai‐Kato et al. (2012)
*s*_0_	Background light attenuation coefficient	m^−1^	0.5	0.08	2	Genkai‐Kato et al. (2012)
*T*	Day length	h	12	4	20	
*v*	Phytoplankton sinking rate	m·d^−1^	0.53	0.033	1.6	Sommer (1984)
$\bar{z}$	Mean depth	m	10	1	100	
*z*_m_	Maximum depth	m	= $\bar{z}/D$			
*θ*	Phytoplankton C:P ratio by atoms	mol·mol^−1^	307	95	519	Elser et al. (2000)
*μ*	Maximum growth rate of phytoplankton	d^−1^	0.76	0.6	0.97	Sommer (1989)

In a shallow lake with a large surface area, *z*_e_ calculated from Equation (3) can be greater than the maximum depth of the lake (*z*_e_ > *z*_m_). In such a case, *z*_e_ was set to *z*_m_. Inputs of phosphorus are external loading from the watershed and release from dead phytoplankton. Losses of phosphorus are due to sequestration in phytoplankton and flushing. The steady‐state solutions for Equations (1) and (2) are

(4) $$N^{*}=\frac{k\left(g+v/z_{e}+h\right)}{\mu ‐g‐v/z_{e}‐h},$$

(5) $$X^{*}=\frac{\left(l‐hN^{*}\right)}{q_{P}\left\{[\mu N^{*}/\left(N^{*}+k)\right]‐eg\right\}}.$$

### 2.2 Lake morphometry

Lake basins are modeled by quadric surfaces, following the approach by Genkai‐Kato and Carpenter (2005). The proportion of the lake's volume that lies above depth *z* is given by

(6) $$V\left(Z\right)=\frac{1}{D}\left[\frac{z}{z_{m}}+\left(3D‐2\right){\left(\frac{z}{z_{m}}\right)}^{2}‐\left(2D‐1\right){\left(\frac{z}{z_{m}}\right)}^{3}\right],$$

where *D* is the ratio of mean depth to maximum depth ($\bar{z}/z_{m}$), called the depth ratio (Carpenter, 1983). The proportion of the lake's volume at depth between *z* and *z* + Δ*z* is calculated as

(7) $$\Delta V=V\left(z+\Delta z\right)‐V\left(z\right)\approx \frac{1}{D}\left[1+2\left(3D‐2\right)\frac{z}{z_{m}}‐3\left(2D‐1\right){\left(\frac{z}{z_{m}}\right)}^{2}\right]\frac{\Delta z}{z_{m}},$$

where Δ*z* is a small, incremental change in depth.

### 2.3 Method 1: Process‐oriented PP model

PP is calculated from the photosynthesis–irradiance relationship. I adopt a minimal relationship between the photosynthetic rate (*P*, mg‐C·mg‐chl^−1^·h^−1^) and light intensity (*I*, µmol·m^−2^·s^−1^):

(8) $$P=P_{\max}\frac{I}{I+I_{k}}$$

Light intensity at depth *z*, at time *t* is calculated using the Beer–Lambert law:

(9) $$I\left(z,t\right)=I_{0}e^{‐(s_{0}+sX)z}\sin\frac{\pi }{T}t.$$

The PP at depth between *z* and *z* + Δ*z*, at time between *t* and *t* + Δ*t* (PP_1_(*z*, *t*), mg‐C) is given by

(10) $${\text{PP}}_{1}\left(z,t\right)=PX^{*}\cdot 10^{6}A\bar{z}\Delta V\cdot \Delta t.$$

The daily areal PP (PP_1_, mg‐C·m^−2^·d^−1^) is given by

(11) $${\text{PP}}_{1}=\sum_{\text{sunrise}}^{\text{sunset}}\sum_{z=0}^{z_{e}}{\text{PP}}_{1}\left(z,t\right)/10^{6}A=\sum_{\text{sunrise}}^{\text{sunset}}\sum_{z=0}^{z_{e}}PX^{*}\bar{z}\Delta V\Delta t.$$

Note that the lake volume (m^3^) and surface area (m^2^) are represented by 10^6^ *A* $\bar{z}$ and 10^6^ *A*, respectively.

### 2.4 Method 2: population dynamics‐based PP calculation

The volumetric PP (PP_2‐vol_, mg‐C·m^−3^·d^−1^) is calculated from the growth term of phytoplankton population dynamics (Equation 1):

(12) $${\text{PP}}_{2\;\text{- vol}}=q_{C}\frac{\mu N^{*}}{N^{*}+k}X^{*}.$$

Because the volume of the epilimnion is represented by 10^6^ *A* $\bar{z}$ *V*(*z*_e_), the daily areal PP in the epilimnion (PP_2_, mg‐C·m^−2^·d^−1^) is given by

(13) $${\text{PP}}_{2}={\text{PP}}_{2\;\text{- vol}}\frac{10^{6}A\bar{z}V\left(z_{e}\right)}{10^{6}A}=q_{C}\frac{\mu N^{*}}{N^{*}+k}X^{*}\bar{z}V\left(z_{e}\right).$$

### 2.5 Parameterization

All parameters are estimated for their default values with their ranges (Table 1). The lake morphometry is represented by lake area (*A*; default: 10 km^2^, range: 0.1–1000 km^2^), mean depth ($\bar{z}$; 10 m, 1–100 m), and depth ratio (*D*; 0.5, 0.33–0.67). The flushing rate (*h*) is estimated by Cole and Pace (1998) and varied over a 10‐fold range. The areal phosphorus inputs (*L*) are based on phosphorus loadings estimated in Lake Mendota, Wisconsin, USA (Lathrop et al., 1998). The volumetric loading rate (*l*) is calculated by $L/\bar{z}$. The phosphorus content of phytoplankton (*q*_P_) is calculated from the phytoplankton carbon content (*q*_C_) in combination with the mean value and standard deviation for the C:P ratios (*θ*) in freshwater systems (Elser et al., 2000): *q*_P_ = 31 *q*_C_/(12*θ*) ≈ 2.58 *q*_C_/*θ*. The light intensity just below the water surface at noon (*I*_0_) is obtained based on the solar irradiance data at Hikone City near Lake Biwa, central Japan (see Appendix S1). The maximum photosynthetic rate (*P*_max_) is assumed to be a function of water temperature (Megard, 1972) and its default, minimum and maximum values are calculated from water temperatures at 20, 10 and 30°C, respectively.

## 3 RESULTS AND DISCUSSION

The phytoplankton density at equilibrium calculated by Equation (5) was 4.9 mg‐chl·m^−1^ when all parameters were set at their default values. Based on the process‐oriented PP model (Method 1) with the default parameter values, areal PP was calculated at PP_1_ = 409 mg‐C·m^−2^·d^−1^. It was calculated at PP_2_ = 332 mg‐C·m^−2^·d^−1^ when the population dynamics‐based PP calculation (Method 2) with the default values was used. The ratio of production in Method 2 to production in Method 1 was 0.81 (i.e., PP_2_/PP_1_ = 332/409 = 0.81). The phytoplankton density and PP calculated from the methods with the default parameter values were within the general ranges of the chlorophyll concentration (2–15 mg‐chl·m^−1^) and the mean primary productivity (250–1000 mg‐C·m^−2^·d^−1^) in mesotrophic lakes (Wetzel, 2001). By this definition, the methods showed that deep lakes tended to be oligotrophic (i.e., <250 mg‐C·m^−2^·d^−1^). The single effect of increased nutrient loading did not result in production typical of eutrophic lakes with its parameter range in either method, but production greater than 1000 mg‐C·m^−2^·d^−1^ was possible when other parameters such as mean depth were simultaneously changed with the nutrient loading rate.

**FIGURE 1:**
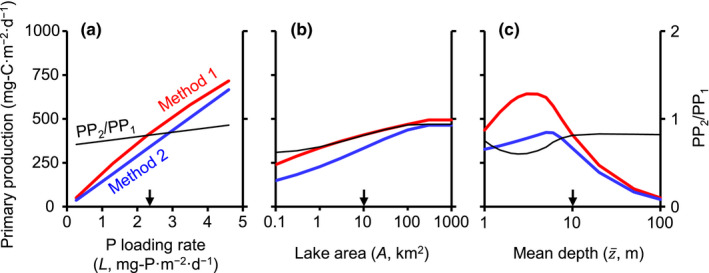
Effects of nutrient loading and lake morphometry on areal primary production calculated by Methods 1 and 2 (left axis). The ratio of production calculated by Method 2 to production calculated by Method 1 (PP_2_/PP_1_) is also indicated (right axis). (a) The effect of areal phosphorus loading rate ($l=L/\bar{z}$). (b) The effect of lake area. (c) The effect of mean depth. Arrows indicate the default values for each *x*‐axis variable

Effects of nutrient loading and lake morphometry on PP are shown in Figure 1. PP was enhanced by increased nutrient loading rate in both methods (Figure 1a). The production slightly increased with lake area (Figure 1b), due to increased epilimnion thickness (Equation 3). The production tended to increase with mean depth when *z*_e_ > *z*_m_ in Equation 3 and therefore *z*_e_ was set to *z*_m_, because increased $\bar{z}$ led to increased *z*_e_; in contrast, it decreased with mean depth when *z*_e_ < *z*_m_ due to a reduction in volumetric nutrient loading rate, $l=L/\bar{z}$ (Figure 1c). The depth ratio had little effect on the production in both methods (see Appendix S3: Figure S2). Variations in nutrient loading and lake morphometry did not result in PP_2_/PP_1_ greater than one.

**FIGURE 2:**
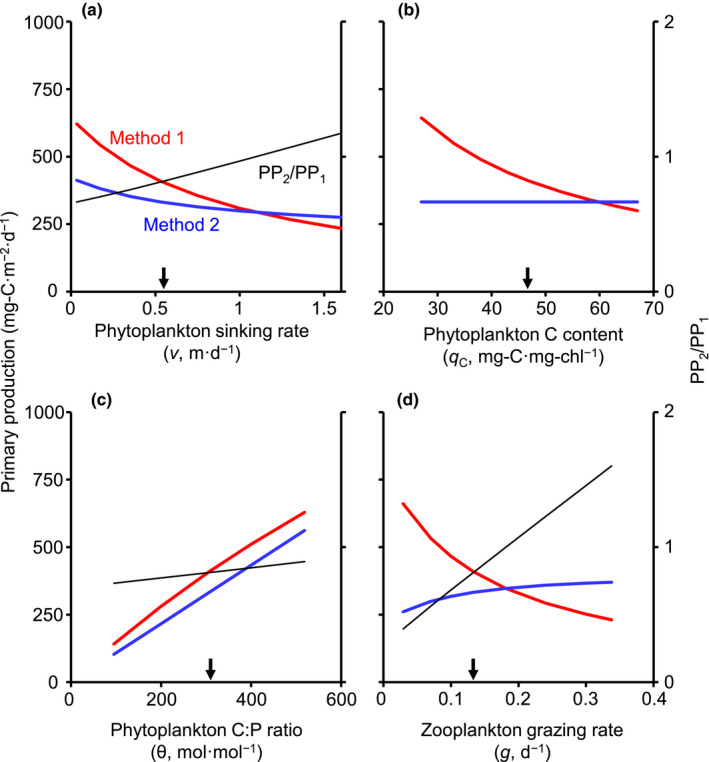
Effects of nutrient‐associated parameters on areal primary production calculated by Methods 1 and 2 (left axis). PP_2_/PP_1_ is also indicated (right axis). (a) The effect of phytoplankton sinking rate. (b) The effect of phytoplankton carbon content. (c) The effect of phytoplankton C:P ratio. (d) The effect of zooplankton grazing rate. Arrows indicate the default values for each *x*‐axis variable

Effects of nutrient‐associated parameters on PP are shown in Figure 2. Increases in the phytoplankton sinking rate (*v*) decreased the production in both methods (Figure 2a), because of decreased algal density in the epilimnion (see Appendix S2: Figure S1). The production decreased with the carbon content of phytoplankton (*q*_C_) in Method 1 and it was independent of *q*_C_ in Method 2 (Figure 2b). This is because an increase in *q*_C_ decreased X* (Equation 11; see Appendix S2: Figure S1), whereas the product of *q*_C_ and X* was a constant (Equation 13). The production was enhanced through increased X* in both methods when the ratio of carbon to phosphorus (*θ*) was raised (Figure 2c; see Appendix S2: Figure S1). The effect of increased grazing rate (*g*) caused an increase in N* and a decrease in X* (see Appendix S2: Figure S1). As a result, the outcomes of the two methods had opposite responses to zooplankton grazing rate: the production decreased with *g* in Method 1, and it increased with *g* in Method 2 (Figure 2d). Effects of other nutrient‐associated parameters on PP were relatively small (see Appendix S3: Figure S3). The PP_2_/PP_1_ ratio took values equal to or greater than 1 when *v* ≥ 1.1 m·d^−1^, *q*_C_ ≥60 mg‐C·mg‐chl^−1^, or *g* ≥ 0.18 d^−1^. Sinking rates (*v*) are related to size and physiological state of algae. High sinking rates are known for large‐sized algae during a stationary or declining phase, compared to small‐sized algae during a phase of rapid increase (Sommer, 1984). Cyanobacteria with gas vesicles and flagellated algae are able to resist sinking (Reynolds, 2006). Colonial algae such as cyanobacteria tend to have high carbon content (*q*_C_), compared to unicellular diatoms and green algae (Riemann et al., 1989). Grazing rates (*g*) have been found commonly to increase with the body size of zooplankton (Peters & Downing, 1984).

**FIGURE 3:**
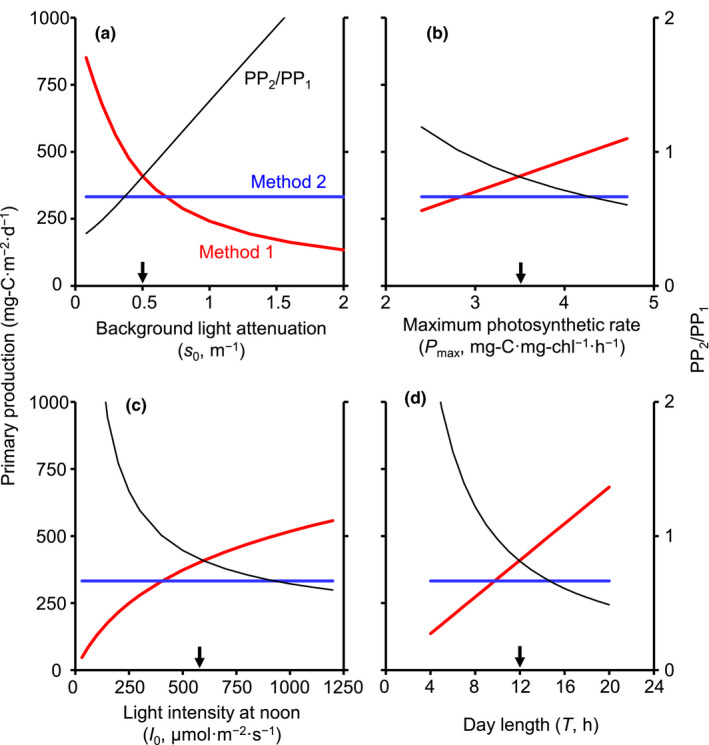
Effects of light‐associated parameters on areal primary production calculated by Methods 1 and 2 (left axis). PP_2_/PP_1_ is also indicated (right axis). (a) The effect of background light attenuation coefficient. (b) The effect of maximum photosynthetic rate. (c) The effect of light intensity at water surface at noon. (d) The effect of day length. Arrows indicate the default values for each *x*‐axis variable

Effects of light‐associated parameters on PP are shown in Figure 3. PP in Method 1 decreased with the background light attenuation coefficient (*s*_0_; Figure 3a), and increased with the maximum photosynthetic rate (*P*_max_; Figure 3b), light intensity just below the water surface at noon (*I*_0_; Figure 3c) and day length (*T*; Figure 3d). The production calculated by Method 2 was not affected by the light‐associated parameters because N* and X* were independent of these parameters. Effects of other light‐associated parameters on PP were relatively small (see Appendix S3: Figure S4). PP_2_/PP_1_ took values equal to or greater than 1 when *s*_0_ ≥ 0.67 m^−1^, *P*_max_ ≤2.85 mg‐C·mg‐chl^−1^·h^−1^, *I*_0_ ≤ 400 µmol·m^−2^·s^−1^, or *T* ≤ 9.75 h. The background light attenuation (*s*_0_) is strongly related to colored compounds (dissolved organic carbon, DOC) and PP has been shown to be considerably decreased by high levels of DOC (Carpenter et al., 1998). PP_2_/PP_1_ could be greater than 1 in winter or in lakes at high latitudes due to a low maximum photosynthetic rate (*P*_max_) under a cold condition. Because the light intensity at noon (*I*_0_) depends on the weather condition, PP_2_/PP_1_ decreases on a sunny day and increases on a rainy day. The effect of photoinhibition at high light intensities, which cannot be incorporated into Equation 8 by a simple modification, is unlikely to be a resolution of the difference between the two methods, because it results only in a downward shift in the depth of the maximum photosynthesis within the water column (see Appendix S4: Figure S5). The default value for day length (*T*) was set at 12 h here, assuming a measurement of annual mean production or production in spring or autumn in temperate lakes. PP_2_/PP_1_ increased with decreased day length, indicating that the difference in production estimates between the methods is reduced in winter (9–10 h of daylight) and is increased in summer (14–15 h) in temperate lakes.

**FIGURE 4:**
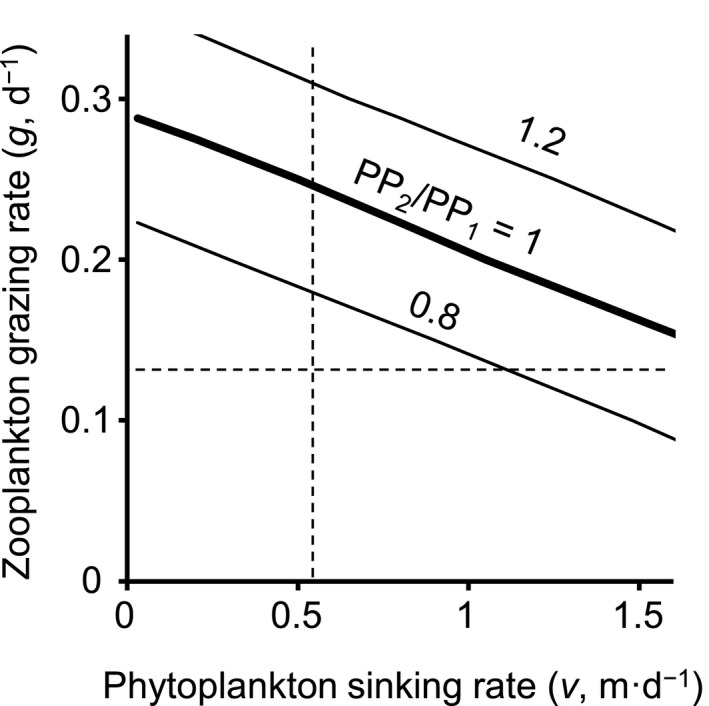
Isopleths for PP_2_/PP_1_ as a function of grazing rate of zooplankton (*g*) and sinking rate of phytoplankton (*v*) under the condition of 15‐h day length. The other parameters were set at their default values. The default values for the grazing and sinking rates are indicated by broken lines

Despite the fundamental difference in the calculation approach, the production estimated from the population dynamics‐based PP calculation (Method 2) was shown to be comparable to, but slightly lower than, that from the process‐oriented PP model (Method 1). The analysis of light‐associated parameters suggests that models of population dynamics in lake ecosystems are likely to be based on an underestimated PP when the models are applied to the growing season of planktonic organisms in spring to summer (i.e., conditions of high water temperature and good light environment). At this time of the year in many mesotrophic and eutrophic lakes, the grazing pressure of large‐sized zooplankton increases due to increased water temperatures and the onset of summer stratification makes large diatoms subject to loss by sedimentation (Kalff, 2002). High grazing rates due to filter feeding crustaceans in spring to summer, compared to the annual mean, have been reported in temperate lakes (Garnier & Mourelatos, 1991; Gulati et al., 1982; Van Donk et al., 1990). In addition, large‐sized phytoplankton accompanying high sinking rates are likely to be dominant under high grazing pressure (Bergquist et al., 1985). Taken together, the production in Method 2 could be comparable to that in Method 1 under the condition of 15‐h day length (Figure 4). Assuming, for example, the grazing rate of 21.5% per day, which is the seasonal mean from May to September in Lake Vechten, the Netherlands (Gulati et al., 1982), PP_2_/PP_1_ fell within the range between 0.8 and 1.2 when the sinking rate of phytoplankton (*v*) took a value between 0.1 and 1.6 m per day (PP_2_/PP_1_ = 1 when *v* = 0.9).

### 3.1 Case for cyanobacteria‐dominant lakes

Later in the growing season due to increased grazing and sinking rates, phytoplankton communities are often replaced by colonial or filamentous cyanobacteria, which are less vulnerable to grazing and sinking (Kalff, 2002). In lakes where cyanobacteria are dominant, the difference in production estimates between the methods would be greater because of low grazing and sinking rates and long daytime, suggesting that the population dynamics model does not represent the actual dynamics of phytoplankton. Because growth of phytoplankton is equivalent in rate to their losses at equilibrium in Equation (1), a reduction in the growth rate of phytoplankton, corresponding to reduced volumetric PP (Equation 12), results from low grazing and sinking rates. Cyanobacteria have been considered as trophic bottlenecks or even dead ends in terms of carbon flow (Fulton, 1988; Havens & East, 1997). Recently, various forms of cyanobacterial mortality such as accidental and regulated cell deaths have been described as a response to biotic and abiotic stresses by molecular, biochemical and morphological studies (Aguilera et al., 2021). There is increasing evidence that the loss processes of cyanobacteria include, to a considerable extent, mortality due to parasitic pathogens such as viruses, bacteria, and fungi (Gerphagnon et al., 2015). Further, cyanobacteria infected by fungal parasites can be food resources directly and indirectly for zooplankton (Agha et al., 2016; Frenken et al., 2018, 2020). As pointed out by Van Donk (1989) that grazing and sinking have been conventionally considered as important loss processes of phytoplankton in models of population dynamics where parasitism has rarely been incorporated as a loss process, the model dealing with cyanobacteria has the potential to enhance its performance by bypassing the trophic bottlenecks. Specifically, another loss term related to parasitic pathogens (bypass) is added to the losses due to grazing and sinking (bottlenecks: small *g* and *v*) in population dynamics of phytoplankton:

(14) $$\frac{dX}{dt}=\frac{\mu N}{N+k}X‐\left(g+d_{p}+\frac{v}{z_{e}}+h\right)X,$$

(15) $$\frac{dN}{dt}=l+q_{P}e\left(g+d_{p}\right)X‐\frac{q_{P}\mu X}{N+k}N‐hN.$$

where *d*_p_ is the death rate related to parasitism. Addition of the parasite‐related death rate to population dynamics of phytoplankton resulted in PP_2_/PP_1_ close to one under the conditions of minimum values for grazing and sinking rates (*g* = 0.029 and *v* = 0.033 in Table 1) and 15‐h day length. For example, PP_2_/PP_1_ was calculated at 1.12 when the parasite‐related death rate was assumed 30% of phytoplankton standing stock (Sigee et al., 2007). Under these conditions, PP_2_/PP_1_ fell within the range between 0.8 and 1.2 when the death rate (*d*_p_) took a value between 0.193 and 0.325 (PP_2_/PP_1_ = 1 when *d*_p_ = 0.26).

## 4 CONCLUSION

The analysis here suggests that models of population dynamics with standard parameterization produce plausible production estimates when the production is measured on an annual timescale in temperate lakes. It is also suggested that the performance of population dynamics in food web and ecosystem models is enhanced and linked closely to empirically derived models by calibrating nutrient‐associate parameters such as grazing and sinking rates when models of population dynamics are applied to specific events on shorter timescales. In lakes with cyanobacterial blooms, model performance would be enhanced by consideration of a source of mortality (e.g., parasite‐related loss) in addition to the losses due to grazing and sinking.

## CONFLICT OF INTEREST

None declared.

## AUTHOR CONTRIBUTIONS

**Motomi Genkai‐Kato:** Conceptualization (lead); Formal analysis (lead); Investigation (lead); Methodology (lead); Resources (lead); Writing‐original draft (lead).

## Supporting information

Appendix S1‐S4

## Data Availability

The computation processes and data necessary to perform the analysis made in this manuscript are archived in Dryad Digital Repository: https://doi.org/10.5061/dryad.ghx3ffbq9.
