# Supplementary material for: Linking population dynamics models with empirically derived models through phytoplankton primary production
Source: Ecol Evol. 2021 Nov 19;11(23):17022–30. doi: 10.1002/ece3.8339 (PMC8668813; doi:10.1002/ece3.8339)
Supplement: Supplementary file 1 — Appendix S1‐S4 [file ECE3-11-17022-s001.docx]

**APPENDICES.** Linking population dynamics models with empirically derived models through phytoplankton primary production.

**Appendix S1. Light intensity just below the water surface at noon based on the solar irradiance data at Hikone City near Lake Biwa, Japan.**

The light intensity just below the water surface measured as photon flux density in units of µmol·m^−2^·s^−1^ was calculated based on the relationships between photon flux densities in the air (*I*_air_) and just below the water surface (*I*_0_) and between photon flux density (*I*_air_) and solar irradiance in units of MJ·m^−2^·h^−1^ (*I*_irradiance_). According to Genkai-Kato et al. (2008), I adopted the following relationships:

*I*_air_ = 488.3 *I*_irradiance_ (S1)

*I*_0_ = *I*_air_ *e*^−0.39^ = 0.677 *I*_air_. (S2)

Combining Equations S1 and S2, the light intensity just below the water surface was expressed as

*I*_0_ = 330 *I*_irradiance_. (S3)

The solar irradiance at Hikone City (35° 16.5' N, 136° 14.6' E), which is located near Lake Biwa, central Japan, was obtained from Japan Meteorological Agency (JMA; http://www.jma.go.jp/). I referred to hourly irradiance data during 11.00 and 13.00 (11.00–12.00 and 12.00–13.00) from 1 January 2018 to 31 December 2019 (730 days). The solar irradiance was 1.94 ± 1.05 MJ·m^−2^·h^−1^ (mean ± SD, *n* = 1459), and maximum and minimum values were 3.75 and 0.08 MJ·m^−2^·h^−1^, respectively. Using Equation S3, the light intensity just below the water surface was calculated as 642 ± 346 µmol·m^−2^·s^−1^, and maximum and minimum values were 1240 and 26 µmol·m^−2^·s^−1^, respectively. Based on this calculation, I set the default, maximum, and minimum values for *I*_0_ at 600, 1200, and 30 µmol·m^−2^·s^−1^ in the text.

**Reference**

Genkai-Kato, M., Onishi, M., Doi, H., Nozaki, K., Yoshino, K., Miyasaka, H., & Omori, K. (2008). Photosynthetic property and primary production of phytoplankton in sublittoral sand bank area in the Seto Inland Sea, Japan. *Ecological Research*, **23**, 1025–1032.

**Appendix S2. Effects of lake morphometry and nutrient-associated parameters on the algal density (*X**) and nutrient concentration (*N**).**

Figure S1. Effects of lake morphometry and nutrient-associated parameters on phytoplankton concentration (left axis) and phosphorus concentration (right axis). Effects of lake area (a), mean depth (b), phytoplankton sinking rate (c), phytoplankton carbon content (d), phytoplankton C:P ratio (e), and zooplankton grazing rate (f). Arrows indicate the default values for each *x*-axis variable.

**Appendix S3. Effects of minor parameters on areal primary production.**

Figure S2. Effects of depth ratio on areal production calculated by Methods 1 and 2 (left axis). PP_2_/PP_1_ is also indicated (right axis). Arrows indicate the default values for each *x*-axis variable.

Figure S3. Effects of nutrient-associated parameters on areal production calculated by Methods 1 and 2 (left axis). PP_2_/PP_1_ is also indicated (right axis). Effects of flushing rate (a), maximum growth rate of phytoplankton (b), half-saturation constant for phytoplankton growth rate (c), and release rate of phosphorus from dead phytoplankton (d). Arrows indicate the default values for each *x*-axis variable.

Figure S4. Effects of light-associated parameters on areal production calculated by Methods 1 and 2 (left axis). PP_2_/PP_1_ is also indicated (right axis). Effects of light intensity at onset of saturation (a) and phytoplankton shading attenuation coefficient (b). Arrows indicate the default values for each *x*-axis variable.

**Appendix S4. Effect of photoinhibition on areal primary production.**

In order to see the effect of photoinhibition under high light intensities, I adopted a minimal model for the photosynthesis–irradiance (P–I) curve according to Genkai-Kato et al. (2008):

*P* = *a I e*^−^*^bI^* (S4)

where *P* represents the photosynthetic rate at a given light intensity (*I*), and *a* and *b* are constants. In Equation S4, *P* is a unimodal function of *I* and takes its maximum value of *P** = *a*/*be* at *I** = 1/*b*. The maximum photosynthetic rate is given by *P*_max_ in the text (i.e., *P** = *P*_max_). Taking these relationships into consideration, the parameters *a* and *b* can be expressed as follows:

*b* = 1/*I** (S5)

*a* = *P*_max_ *b e*. (S6)

Based on the P–I curve given in Equation S4, the relationships between areal primary production and the light intensity at onset of photoinhibition (*I**) are shown in Figure S4a. The production was independent of *I** in Method 2, whereas it decreased with *I** in Method 1. The decreased production in Method 1 can be explained by a downward shift of depth at maximum production with decreased *I** (Figure S4b). Although the production near the water surface was smaller for phytoplankton with smaller *I**, the depth-integrated production was greater due to the subsurface maximum of photosynthesis.

Figure S5. (a) Effect of photoinhibition on areal primary production calculated by Methods 1 and 2 (left axis). PP_2_/PP_1_ is also indicated (right axis). (b) Vertical profiles of daily production at each 0.1 m depth interval with different light intensity at onset of photoinhibition (*I** = 100, 300 and 600 µmol·m^−2^·s^−1^), calculated from Method 1. Parameters were set at their default values.

**Reference**

Genkai-Kato, M., Onishi, M., Doi, H., Nozaki, K., Yoshino, K., Miyasaka, H., & Omori, K. (2008). Photosynthetic property and primary production of phytoplankton in sublittoral sand bank area in the Seto Inland Sea, Japan. *Ecological Research*, **23**, 1025–1032.
